# A Case of Urinary Tract Infection and Severe Sepsis Caused by* Kluyvera ascorbata* in a 73-Year-Old Female with a Brief Literature Review

**DOI:** 10.1155/2017/3848963

**Published:** 2017-05-03

**Authors:** Majd Alfreijat

**Affiliations:** Department of Medicine, St. Joseph's Hospital and Medical Center, Phoenix, AZ, USA

## Abstract

Infections that are caused by* Kluyvera* bacteria have been previously reported in the medical literature; however, they seem to be less common. Herein, we report a case of urinary tract infection and severe sepsis caused by* Kluyvera ascorbata* in a 73-year-old female. We also did a brief literature review of infections caused by this organism in adults.

## 1. Introduction

*Kluyvera* is a Gram-negative bacterium that belongs to the Enterobacteriaceae family. Infections caused by this organism are not very common; however, they have been previously reported in the literature. Herein, we report a case of severe sepsis in a 73-year-old female that was a result of urinary tract infection due to* Kluyvera ascorbata*.

## 2. Case Presentation

73-year-old female with history of hypertension, and a recent diagnosis of right lower lobe (RLL) lung mass, presented to the emergency department (ED) complaining of progressively worsening dysphagia for few weeks. The patient had difficulty in swallowing both liquids and solids. She reported decreased appetite, weight loss, and multiple episodes of nonbloody vomit. She was scheduled to have an endoscopy as an outpatient on the same day of admission; however, she decided to come to the ED instead, as her symptoms became more severe.

The patient had a remote history of 20 pack-year of cigarettes smoking. Her medications included Aspirin, Iron Pills, Atorvastatin, and Lisinopril. On physical exam, she was hypothermic with a temperature of 35.6 degrees Celsius and tachycardic with a heart rate of 103. The patient looked cachectic and chronically ill. She also had dry oral mucous membranes. The initial laboratory results showed significant leukocytosis with a white blood cell count of 29.3 thousand/ul and hyponatremia with a sodium of 127 mmol/L. The urine was cloudy in appearance, and it contained leukocytes esterase and more than 50 white blood cells. The chest X-ray revealed RLL mass, with no evidence of pulmonary consolidation. The patient was admitted to a telemetry bed and was started on aggressive IV hydration and IV ceftriaxone as a treatment for severe sepsis syndrome. The source of the sepsis was thought to be a urinary tract infection as the urine culture grew* Kluyvera ascorbata* and* Streptococcus agalactiae* (group B), and both organisms were sensitive to ceftriaxone. The hospital course was prolonged and complicated. During the first 24 hours, the patient went into atrial fibrillation with rapid ventricular rate and was started on IV amiodarone and heparin drip. She also had an abrupt onset of cold and discolored left leg that required an emergent vascular surgery evaluation. The patient was found to have left femoral artery occlusion and underwent left iliac artery stenting and left femoral endarterectomy.

Furthermore, she had a CT-guided biopsy of the RLL mass and the pathology was consistent with primary adenocarcinoma. The patient was intubated for the surgical procedure and the biopsy. Few days later, she was briefly extubated; however, she continued to have significant hypoxia and hypotension. There was also a concern for aspiration pneumonia. Palliative care team was involved and after an extensive discussion with family, the decision was taken to change the code status to DNR. Patient expired after 2 weeks of hospitalization. The endoscopy was never done as the patient was never in a stable condition throughout her hospital stay.

## 3. Discussion

*Kluyvera* is a group of Gram-negative rods bacteria and a member of the family Enterobacteriaceae [1]. It was named after Albert Jan Kluyver, a prominent Dutch microbiologist who was the first to propose its existence in 1936 [2]. The organism was isolated from soil and sewage by Asai et al. in 1956 [3]; however, it was not until 1981 that its molecular characteristics were defined by Farmer et al. [4].* Kluyvera* genus has four species:* Kluyvera ascorbata, Kluyvera cryocrescens,* and* Kluyvera georgiana* that were all found in humans and* Kluyvera cochleae* that was isolated from snails and slugs [5].

Between 1980 and 2005 there have been 41 cases of clinically significant infections in humans caused by* Kluyvera*: 21 of them were due to* K. ascorbata*, 8 due to* K. cryocrescens*, and 12 were due to an unspecified* Kluyvera* [6].

In 2001 Sarria et al. published the largest retrospective analysis about the clinical manifestations of infections caused by* Kluyvera* and that included gastroenteritis (diarrhea and fever), acute pancreatitis, bacteremia, wound infection, urinary tract infection (UTI), pyelonephritis, acute cholecystitis, peritonitis, mediastinitis, infected urethrorectal fistula, and soft tissue infection [7]. Since 2005, and to the best of our knowledge, there have been only 6 other cases of* Kluyvera ascorbata *infection in adults manifested as UTI, pyelonephritis, sepsis, liver abscess, and bacteremia [8–12]. Interestingly, in one of these cases the bacteria were multidrug resistant [11].

## 4. Conclusion

It is essential to report common conditions like UTIs that are caused by less common microbes like* Kluyvera ascorbata*. This could help in raising awareness of this organism in the medical society.
